# Revisiting the Extinction of the RNA World

**DOI:** 10.1021/acs.biochem.2c00121

**Published:** 2022-04-07

**Authors:** Anthony C. Forster

**Affiliations:** Department of Cell and Molecular Biology, Uppsala University, Husargatan 3, Box 596, Uppsala 75124, Sweden

## Abstract

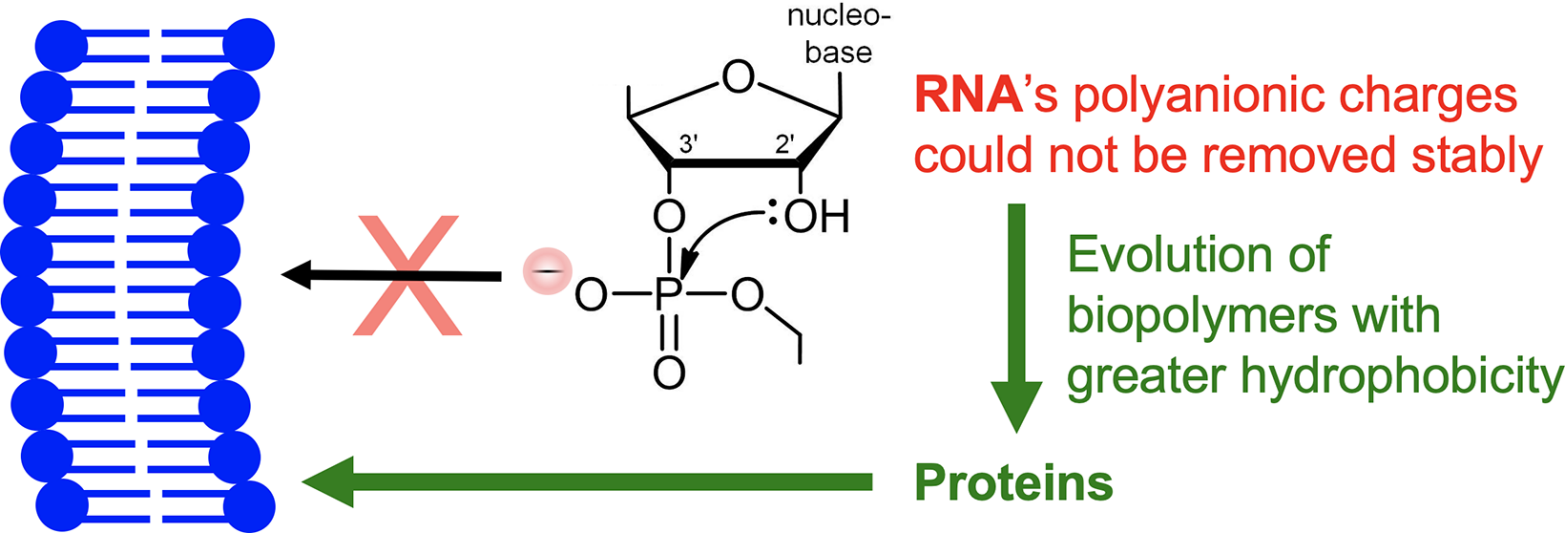

The ribozyme world is thought to have evolved the burdensome complexity of peptide and protein synthesis because the 20 amino acid side chains are catalytically superior. Instead, I propose that the Achilles heel of the RNA world that led to the extinction of riboorganisms was RNA’s polyanionic charges that could not be covalently neutralized stably by phosphotriester formation. These charges prevented development of hydrophobic cores essential for integration into membranes and many enzymatic reactions. In contrast, the phosphotriester modification of DNA is stable. So, the fact that the charge was never removed in DNA evolution gives further credence to proteins coming before DNA.

In 1962, Alexander Rich published a revolutionary paper that was decades ahead of its time and is still underappreciated. In pondering whether genes (nucleic acids) or enzymes (proteins) came first in life, he concluded that the first gene and biomolecular catalyst were both RNA.^1^ Although he did not propose efficient ribozymes, their subsequent discoveries and the identification of an RNA active site in the ribosome (the complex of 50+ proteins and RNAs that synthesizes proteins) led to wide acceptance of an RNA world.^2^ The idea naturally raised two further questions:

(i) Why did DNA and protein evolve?
(ii) Which came next after RNA: DNA or protein? (See green arrows in Figure 1.)

**Figure 1:**
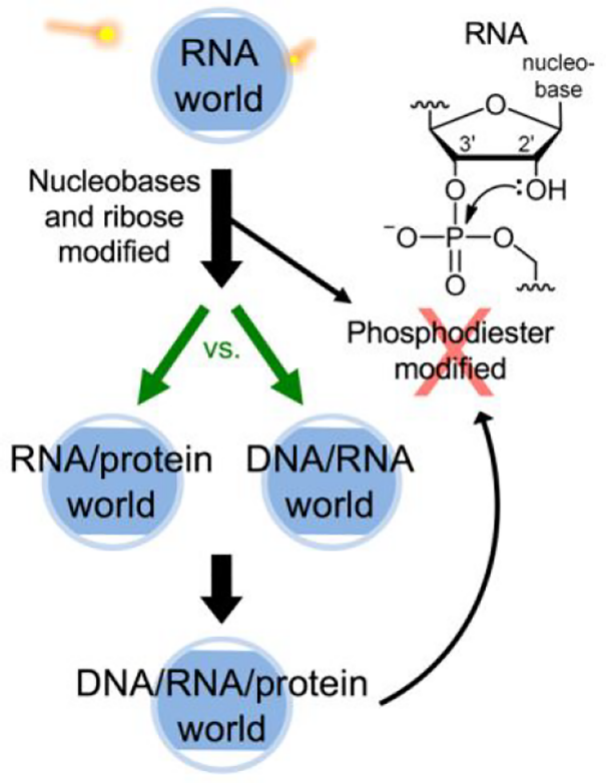
Evolution of RNA to encode polymers containing hydrophobic cores. Two easy evolutionary roads to hydrophobic cores were not taken by RNA (two arrows to red X) because the removal of the negative charge shown leads to rearrangement. Rearrangement is inhibited in DNA by its 2′ H, yet DNA also did not lose charges, giving further credence to protein evolving first (left green arrow).

Let us take question (i) first because there is general agreement on the answer.^2^ Compared with RNA’s susceptibility to base-catalyzed cleavage via transesterification to its vicinal 2′ hydroxyls (Figure 1, right) and the chemical limitations of its four inert nucleobases, DNA is stable due to the 2′ deoxyribose modification, while protein enzymes are not only stable but also catalytically much more versatile due to sporting 20 different amino acid side chains.^3^ These are very reasonable answers chemically (although even the 20 amino acid side chains are far from perfect as they lack an electrophile and protein enzymes rely heavily on cofactors including metals and also post-translational modification). But is this side-chain-diversity explanation unassailable?

Consider that the list of more than 110 RNA modifications (https://genesilico.pl/modomics/) contains many hypermodifications that include most of the catalytic groups of proteins. Admittedly, only 18 mostly simple modifications are found in all three domains of life: eubacteria, archaebacteria, and eukaryotes.^4^ Of these, the carboxylic acid of t^6^A is the only significant catalytic-group-like modification, but it should not have been hard to evolve others. For example, additional nucleotide modifications with catalytic functionality were argued to be part of the RNA world based on the universal coenzymes with nonfunctional short RNA handles: NAD^+^, *S*-adenosylmethionine, CoA, FAD, and ATP.^5^ This implies that the RNA world contained ribozymes catalyzing redox, transmethylation, C–C bond formation, and phosphorylation reactions.^5^ So, perhaps the Achilles heel of ribozymes was not side-chain diversity after all. Instead, might it have been the polyanionic backbone?

The highly charged nature of RNA would have posed two big challenges for the RNA world: synthesizing membrane pores/transporters/scaffolds^6^ (although small-molecule pores are possible^7^) and synthesizing enzymes with hydrophobic active sites. Charged groups prevent integration into membranes. Hydrophobic cores are crucial for protein enzymes catalyzing reactions that are susceptible to hydrolysis or involve highly reactive intermediates (e.g., the carbon-based radicals necessary for biochemical synthesis of all deoxyribonucleotides, reactions presumed by some to be incompatible with ribozymology^8,9^), although nucleic-acid-based hydrophobic pockets have been demonstrated^10^ and hydrophobic base modifications could assist their formation.^11^ Life’s ultimate solution of encoding polymers with an uncharged backbone by evolving the complexity of peptide^12,13^ and then protein synthesis^14^ seems much more convoluted (Figure 2) and metabolically burdensome to the cell than the alternative of inventing synthesis of less-charged (or uncharged) modified RNAs.

**Figure 2:**
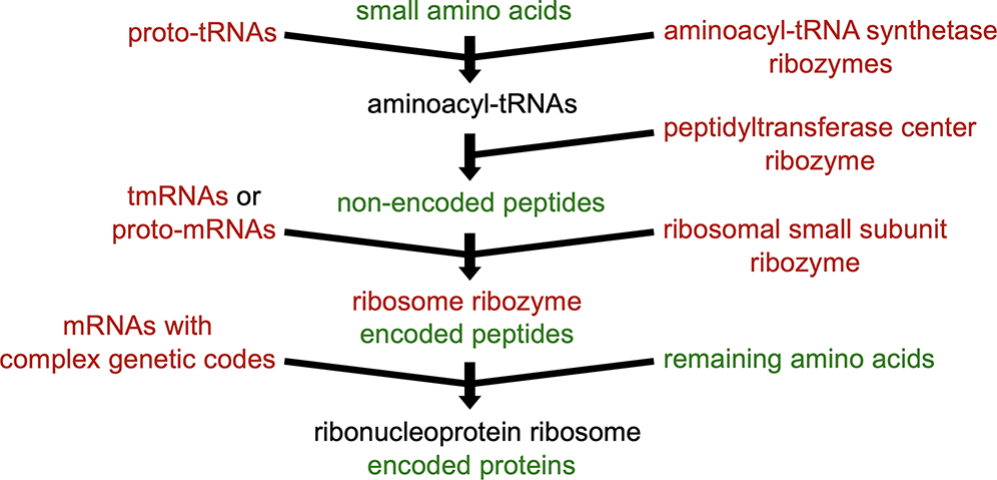
Evolution of protein synthesis. The proposed pathway from amino acids to proteins (green) is based on RNAs (red). Identifiable descendants of aminoacyl-tRNA synthetase ribozymes are extinct, and the ancestral function of the ribosomal small subunit ribozyme is a mystery. Although this pathway is necessarily very complex and speculative, many^14,15^ of the pathways proposed in the literature are similar. A quite different alternative is evolution from an RNA helicase.^16^

Conceivably, less-charged (or uncharged) RNAs might have been synthesized directly from nucleic acid templates using appropriate nucleotide analogue substrates, although RNA’s charge is very important for aqueous solubility and specific double-helical structures longer than several base pairs^17^ (as learned by the antisense drug companies; peptide nucleic acid^13^ is an exception). An alternative is synthesis by post-transcriptional modification of RNA by alkylation or acylation. Although the phosphodiester O^–^ is relatively inert (its alkylation is avoided even in small-molecule phosphate metabolism), the O^–^ is more reactive than most other groups of nucleic acids that become alkylated.^18,19^ The coexistence of two types of RNA polymers could potentially create specificity problems in the cell, but today’s modified RNAs do not interfere with the template functions of unmodified RNAs and vice versa. So, why did RNA not take this short evolutionary path (Figure 1, top right)?

A clue comes from examining evolutionary tinkering of the chemical groups of RNA (https://genesilico.pl/modomics/). Interestingly, the phosphodiester is the only chemical group of RNA frozen in evolution: metabolism does not modify even a single one. (However, there is a phosphodiester modification in DNA: phosphorothioate.^20^) The reason, presumably, is that the delocalized negative charge on the phosphodiester group stabilizes it against nucleophilic attack.^3^ Removal of this charge in RNA makes the phosphorus more susceptible to nucleophilic attack by the vicinal 2′ hydroxyl group (Figure 1, right). Indeed, such phosphotriester products in RNA are intrinsically unstable under physiological conditions, although they can be stabilized somewhat by certain branches.^21,22^ RNAs with the conserved 2′-O-methylation modification would enable a modification at the adjacent phosphodiester to give a stable neutral backbone, but this is also unseen in nature, perhaps because it requires double tinkering.

The lability of RNA phosphotriester modifications not only explains why RNA did not shed any negative charges and why peptide and protein synthesis evolved but also may bear on question (ii) above. This question was not resolved by comparative genomic and structural analyses.^9^ The aforementioned biochemical synthesis of deoxyribonucleotides via free radicals (which degrade RNA) has been interpreted as favoring late DNA.^8,9^ Proposed alternative chemical routes to deoxyribonucleotides^9,23,24^ that are much simpler than evolving a ribosome^14^ favor early DNA. Now consider the fact that phosphotriester groups are much more stable in DNA than RNA courtesy of the 2′ deoxy modification.^18,19,22^ Thus, if DNA came before protein in life,^5,9,23^ it would seem reasonable that evolution would have crossed a shorter evolutionary distance (versus inventing peptide and protein synthesis) to invent DNAzymes^25^ that included phosphodiester charge removal to enable large hydrophobic cores. This could be tested by generating such modified DNAzymes in the lab, and they might be useful as therapeutics. But given that this pathway did not evolve, this gives further credence to proteins coming before DNA to give rise to a ribonucleoprotein world^8^ (Figure 1, left).

With regard to enzyme evolution, to paraphrase Frank Westheimer,^3^ an explanation of ultimately why nature did not choose phosphates is their unsuitability for creating hydrophobic cores. It is very plausible that the inability of modified RNAs to shed negative charge stably was the evolutionary baggage that led to the extinction of the RNA world, the mother of all extinctions.

## Abbreviations

ATP: adenosine triphosphate
CoA: coenzyme A
FAD: flavin adenine dinucleotide
NAD+: nicotinamide adenine dinucleotide
t^6^A: *N*^6^-threonylcarbamoyladenosine
